# Psychosocial aspects of rosacea: patient-reported coping strategies in Saudi Arabia

**DOI:** 10.3389/fmed.2025.1634437

**Published:** 2025-09-22

**Authors:** Faten Albukhari, Kayan Alotaibi, Ebtesam Almajed, Wijdan A. AlMutiri, Nuwayyir Abdullah Alqasimi, Nouf Abdullah Alzahrani, Hayat Alzahrani

**Affiliations:** 1 Department of Internal Medicine, College of Medicine, Princess Nourah bint Abdulrahman University, Riyadh, Saudi Arabia; 2 Clinical Science Department, College of Medicine, Princess Nourah bint Abdulrahman University, Riyadh, Saudi Arabia; 3 Department of Family and Community Medicine, College of Medicine, Princess Nourah bint Abdulrahman University, Riyadh, Saudi Arabia

**Keywords:** rosacea, coping strategies, brief-COPE, Saudi Arabia, problem-focused coping

## Abstract

**Background:**

Rosacea is a chronic inflammatory skin condition that negatively impacts patients’ psychological well-being. Coping strategies can influence disease adaptation and psychosocial outcomes. However, little is known about how rosacea patients in Saudi Arabia cope with the burden of this disease.

**Methods:**

A cross-sectional study was conducted using a structured tool that combined clinical assessments with the Arabic version of the Brief COPE. Participants were recruited through dermatology clinics and online platforms. Coping strategies were categorized into three domains: problem-focused, emotion-focused, and avoidant. Statistical analyses included correlation tests and post-hoc clustering.

**Results:**

176 participants (mean age: 28.8 ± 8.25 years; 79.5% female) completed the survey. Problem-focused coping had the highest mean score (68.48), followed by emotion-focused (60.98) and avoidant strategy (49.01). Maladaptive strategies, such as substance use and behavioral disengagement, were the least adopted. Significant positive correlations were observed between disease duration and all three coping styles, with the strongest correlation found for the emotional-focused coping strategy (r = 0.323, *p* < 0.001). Higher rosacea severity was positively associated with a longer disease duration (r = 0.317, *p* < 0.001). Higher education as well as employment status were correlated with greater use of adaptive strategies, while students scored the lowest across all domains.

**Conclusion:**

Rosacea patients in Saudi Arabia predominantly use constructive, culturally grounded coping strategies, with religion playing a crucial role. Sociodemographic factors such as education and employment significantly influence coping profiles. In light of these findings, it is essential to develop culturally sensitive interventions that promote adaptive coping and integrate social and spiritual support.

## 1 Introduction

*Rosacea* is a chronic, progressive, inflammatory dermatological condition characterized by a wide range of clinical features, including facial erythema, telangiectasias, papules, and pustules (1). Globally, rosacea affects approximately 5.6% of adults, with a reported prevalence of 2.39% among patients attending dermatology clinics. Both women and men are affected by rosacea, with the majority of patients falling between the ages of 45 and 60 (2). In Saudi Arabia, the prevalence is estimated at 1.59%, as reported by Al-Hoqail (3).

Despite extensive research, the pathogenesis of rosacea remains unclear. Some of the proposed etiopathogenic factors include genetic susceptibility, immune dysregulation, and increased activity of transient receptor potential cation channels, resulting in elevated levels of vasoregulatory neuropeptides responsible for the flushing associated with rosacea. Additional contributors include UV radiation, psychogenic factors, and skin commensals, such as *Staphylococcus epidermidis* and Demodex species (5). Clinically, rosacea follows a relapsing–remitting course and is classified into four main subtypes by the National Rosacea Society: erythematotelangiectatic, papulopustular, phymatous, and ocular (6).

In societies that promote youthful, flawless, and glowing smooth complexions, the perception of being different and the feeling of inferiority due to one’s appearance contribute to existential distress, subsequently arousing negative attitudes towards oneself, the condition, and society in general. The distress experienced by rosacea patients paradoxically intensifies the symptoms, thereby negatively impacting their general health and emotional well-being, which further exacerbates their distress and negative attitudes. A review of the medical literature examining the psychiatric comorbidities and psychological distress associated with rosacea revealed that rosacea patients are more likely to suffer from depression, low confidence/self-esteem, social anxiety, stress, as well as decreased quality of life (7–11).

A growing body of literature highlights the psychiatric comorbidities associated with rosacea, including depression, anxiety, low self-esteem, stress, and reduced quality of life. Rosacea is increasingly recognized not only as a chronic inflammatory skin disorder but also as a condition with substantial psychosocial consequences. An international study conducted in eight countries (Germany, the UK, Ireland, Sweden, Denmark, France, Italy, and Mexico) that examined the influence of facial erythema on psychological well-being in a sample of 6,831 participants found that facial erythema was strongly related to poor health status and negative personality traits. Rosacea has adverse psychological, social, and occupational effects. Approximately 80% of respondents also reported difficulty controlling facial erythema (12).

Coping is defined as “a cognitive behavioral effort to master, reduce, or tolerate the internal and/or external demands that are created by the stressful transaction” (13). Johnston et al. examined the experience of living with rosacea and of seeking and receiving treatment. The study found that participants employed emotion-focused and behavioral/avoidant-focused coping strategies to navigate the adverse effects of rosacea on their lives (14).

Several studies have documented significant impairment in quality of life, with coping strategies often centered on instrumental social support and closely linked to demographic factors such as age, gender, and employment status (4). In male patients, long-term disease was associated with high psychosocial distress, yet the uptake of coping mechanisms remains low, highlighting a gender-specific support gap (15). Other research indicated a predominance of emotion-focused avoidance coping strategies in rosacea, which may exacerbate psychological distress (16). Psychosocial impacts include increased rates of depression, anxiety, social withdrawal, and stigmatization, with evidence that effective treatment can improve both clinical symptoms and psychological well-being (17, 18). Compared to psychiatric cohorts, rosacea patients have demonstrated a higher sense of coherence and tend to employ more problem-solving and support-seeking strategies (19). Qualitative accounts further reveal that the visible severity of the condition does not always correlate with perceived burden, underscoring the need for individualized, patient-centered psychosocial interventions (20).

Upon reviewing the literature, despite the global recognition of rosacea’s psychosocial consequences, studies investigating coping strategies among rosacea patients in Saudi Arabia remain limited. The failure of patients to select appropriate stress-coping strategies and heightened emotional reactivity could intensify symptoms and impede treatment outcomes. To optimize rosacea treatment, patients’ emotional and psychological well-being should be considered to facilitate healthy coping strategies. Therefore, to gain an in-depth understanding of the experiences of patients with rosacea, this study aimed to assess the disease coping strategies of patients with rosacea in Saudi Arabia and to guide healthcare professionals toward more comprehensive management that addresses the condition’s physical and psychological dimensions.

## 2 Methods

### 2.1 Study design and population

An anonymous cross-sectional study using a convenience non-probability sampling technique was conducted at the dermatology outpatient clinics of a single institution. In addition to clinic-based recruitment, questionnaires were distributed electronically to the broader Saudi population through social media platforms, employing the same sampling strategy.

Eligible participants were adult residents of Saudi Arabia (aged 18 years or older) who had been diagnosed with rosacea by a dermatologist. Participants were required to provide electronic informed consent and complete the questionnaire thoroughly. Respondents under 18 years of age were excluded. The sample size was calculated using G-Power version 3.1, based on the primary objective of assessing correlations between coping strategy scores and clinical or sociodemographic variables. For a two-tailed Pearson’s correlation test with a small-to-moderate anticipated effect size (r = 0.25), α = 0.05, and statistical power = 0.80, the minimum required sample size was 123 participants. Recruitment exceeded this number to compensate for potentially incomplete or inaccurately completed questionnaires. Our final sample of 178 participants exceeded this requirement, ensuring sufficient power for the planned analyses.

### 2.2 Ethics statement and informed consent

Ethical approval was obtained from the Institutional Review Board (IRB) of Princess Nourah bint Abdulrahman University (No. 23–0510) prior to study initiation. All participants were provided with clear explanations regarding the study’s purpose, procedures, and their rights. Electronic informed consent was obtained before survey completion. Participation was voluntary and anonymous, and participants retained the right to withdraw from the study at any point without consequences. Confidentiality was maintained by anonymizing data through numeric coding, preventing individual identification.

### 2.3 Data collection procedure and study survey

Data were collected via a Google Forms survey accessible through a QR code. Recruitment was conducted through: (1) direct approach of dermatology clinic patients with a dermatologist-confirmed diagnosis of rosacea, and (2) online invitations via social media platforms. Using both methods allowed access to a broader and more diverse population. To minimize potential selection bias, both recruitment groups received the same questionnaire format and instructions.

The questionnaire was designed to gather comprehensive information through four structured sections: sociodemographic characteristics, clinical details, rosacea severity, and coping strategies. Prior to data collection, the questionnaire was critically reviewed by three dermatology experts for content accuracy and clarity and then piloted on a non-medical sample to ensure readability and acceptability. The final questionnaire was translated into Arabic by four native speakers, subsequently verified by back-translation, and approved by two dermatologists.

Participants provided data on age, gender, nationality, region of residence, marital status, educational level, employment status, monthly family income, and presence of chronic illnesses (e.g., diabetes, hypertension, asthma, cardiovascular diseases). Clinical data included dermatologist-confirmed rosacea diagnosis, disease duration, current treatment modalities, sunscreen use frequency, and past use of unauthorized topical bleaching creams. Rosacea severity was self-assessed using validated items developed by Seo et al. (21), with slight modifications made for local relevance. The Clinician’s Erythema Assessment (CEA), area, and telangiectasia (CAT) scores assess severity and treatment efficacy. The CEA is a five-point scale ranging from clear (0) to severe (4). The Area Score uses the palm method on a three-point scale to determine erythema extent. The telangiectasia severity score is also measured on a three-point scale. The total CAT score was calculated by summing these three subscores, with higher scores indicating more severe disease. Representative photographs and detailed scoring instructions were included in the survey to help participants accurately self-assess. The CAT tool has demonstrated validity and reliability. Additional items adapted from Tuchayi et al. (22) were included to evaluate rosacea severity by examining symptoms of papulopustular, phymatous, and ocular rosacea. To ensure consistency across recruitment methods, both in-person and online participants received the same version of the questionnaire. Detailed descriptions and representative visual scales were included in the questionnaire to help participants accurately score their symptoms.

Coping strategies were assessed using the Arabic version of the Coping Orientation to Problems Experienced Inventory (Brief-COPE) (23, 24), a validated tool that measures both effective and ineffective coping responses to stress. The Brief-COPE includes 28 self-report items scored on a Likert scale (1 = “I have not been doing this at all”; 4 = “I have been doing this a lot”). Scores were calculated as averages for each coping domain, offering a continuous measure of the degree to which an individual engages in that particular coping style. Three overarching coping domains were derived from grouped subscales: problem-focused coping (Items 2, 7, 10, 12, 14, 17, 23, 25), emotion-focused coping (Items 5, 9, 13, 15, 18, 20, 21, 22, 24, 26, 27, 28), and avoidant coping (Items 1, 3, 4, 6, 8, 11, 16, 19). In addition to domain-level scores, the Brief-COPE offers insights into 14 individual coping facets, each assessed by two items. These include: active coping (Items 2, 7), instrumental support (Items 10, 23), positive reframing (Items 12, 17), planning (Items 14, 25), emotional support (Items 5, 15), venting (Items 9, 21), humor (Items 18, 28), acceptance (Items 20, 24), religion (Items 22, 27), self-blame (Items 13, 26), self-distraction (Items 1, 19), denial (Items 3, 8), substance use (Items 4, 11), and behavioral disengagement (Items 6, 16).

### 2.4 Statistical analysis and data management

Statistical analysis was conducted using R version 4.3. Continuous variables were summarized using means ± standard deviations or medians and interquartile ranges, depending on their distribution. Categorical variables were presented as frequencies and percentages. The independent sample t-test was used to assess the associations between coping strategies and continuous sociodemographic or clinical variables (e.g., age, disease duration). Differences in mean scores across the 14 Brief-COPE subscales and the three domains were evaluated using the paired t-test with false discovery rate correction. Compact letter displays were used to indicate statistically homogeneous groups; means not sharing a letter were considered significantly different (*p* < 0.05). Chi-square tests examined associations between coping strategies and categorical variables (e.g., gender, rosacea subtype). Pearson’s correlation coefficient was used for normally distributed continuous variables, and Spearman’s correlation for non-normally distributed data. Missing data were imputed as appropriate. Hypothesis testing was conducted at a 5% level of significance. All survey responses were complete, and no missing data were encountered for any study variable.

## 3 Results

A total of 176 participants completed the questionnaire, with a mean age of 28.8 ± 8.25 years. The majority of the sample were female (79.5%, *n* = 140), while males accounted for 20.5% (*n* = 36). Most participants were Saudi nationals (84.1%, *n* = 148). Geographically, the highest proportion resided in the Central region (48.9%, *n* = 86), followed by the Western region (22.2%, *n* = 39) and the Eastern region (10.8%, *n* = 19). Regarding marital status, 57.4% (*n* = 101) of the respondents were single, and 39.8% (*n* = 70) were married. Most participants held a bachelor’s degree (68.8%, *n* = 121), while 18.2% (*n* = 32) had attained a high school education or less. Regarding employment status, 38.1% (*n* = 67) were employed, 27.3% (*n* = 48) were students, and 18.2% (*n* = 32) were unemployed. Nearly half of the respondents (48.3%, *n* = 85) reported a monthly income exceeding 10,000 SAR. Table 1 presents the sociodemographic characteristics of the studied sample.

**Table 1:** Descriptive analysis of the study sample (*N* = 176).

Variables	Mean (SD)
Age	28.8 (8.25)
	*N* (%)
Gender	
Female	140 (79.5%)
Male	36 (20.5%)
Nationality	
Non-Saudi	28 (15.9%)
Saudi	148 (84.1%)
Place of residence	
Central region	86 (48.9%)
Eastern region	19 (10.8%)
Northern region	15 (8.52%)
Southern region	17 (9.66%)
Western region	39 (22.2%)
Marital status	
Divorced	2 (1.14%)
Married	70 (39.8%)
Single	101 (57.4%)
Widowed	3 (1.70%)
Education	
Bachelor’s degree	121 (68.8%)
High school or less	32 (18.2%)
Postgraduate	23 (13.1%)
Occupation	
Employed	67 (38.1%)
Housewife	29 (16.5%)
Student	48 (27.3%)
Unemployed	32 (18.2%)
Monthly income	
<5,000	38 (21.6%)
5,000–10,000	53 (30.1%)
>10,000	85 (48.3%)

Maladaptive coping strategies, such as using alcohol or drugs, were the least commonly endorsed, with 95 and 94% of respondents stating they had not engaged in these behaviors at all. In contrast, adaptive problem-focused strategies were more frequently used: “Taking action to try to make the situation better” was practiced to a moderate or great extent by 70% of participants, followed by “Trying to come up with a strategy about what to do” (62%) and “Thinking hard about what steps to take” (68%).

Approximately two-thirds of participants (64%) reported “trying to get advice or help from others,” and 54% indicated that they sought “help and advice from other people” to a moderate or large extent. Religious or spiritual coping was less prevalent, with only 33% reporting minimal engagement (not at all/a little bit), while 62% practiced it to a moderate or great extent. Additionally, 60% of respondents reported frequently using the strategy “saying to myself this is not real,” and 56% reported “saying things to let my unpleasant feelings escape” to a similar extent.

Behaviors such as “giving up trying to deal with it” and “giving up the attempt to cope” were endorsed to a moderate or high extent by 31 and 29% of participants, respectively. Overall, the most frequently utilized coping strategies were adaptive or problem-focused, whereas the least commonly used strategies were avoidant or potentially harmful, such as substance use.

**Figure 1:**
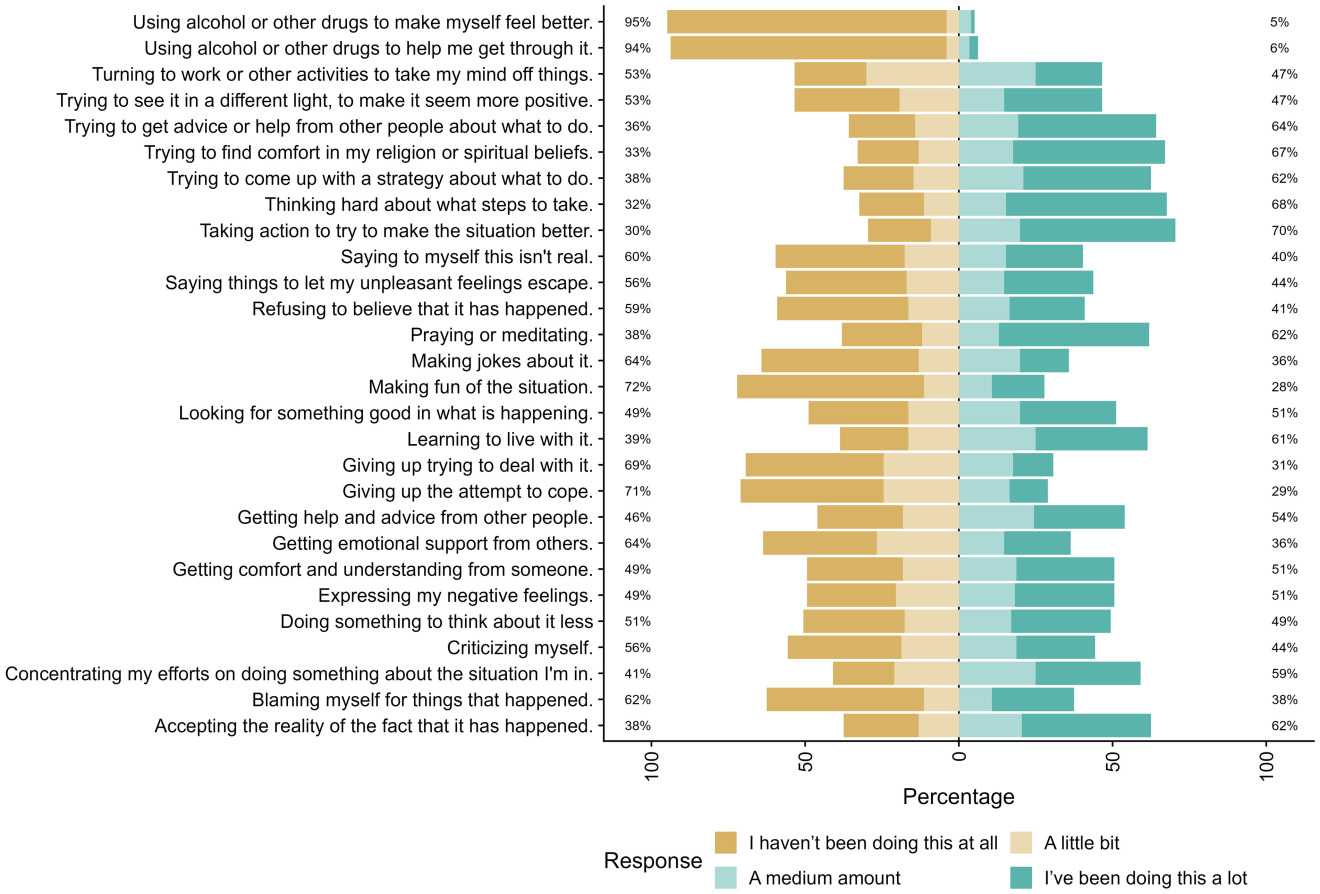
Distribution of coping strategy usage among study participants. Distribution of participants’ responses to the 28 items of the Brief-COPE questionnaire. Each bar represents the percentage distribution of responses across four usage levels: “I have not been doing this at all” (dark brown), “A little bit” (light brown), “A medium amount” (light teal), and “I have been doing this a lot” (dark teal). Percentages on the left indicate the proportion of participants who reported no use of each strategy, while percentages on the right show the combined moderate-to-high usage rates.

The median Clinical Erythema Assessment (CEA) score was 5.00 [IQR: 3.00–7.00] (Table 2). The majority of participants were classified as papule grade 1 (64.8%, *n* = 114), followed by grade 2A (23.9%, *n* = 42). Higher severity grades, such as 3B and 4C, were less frequently observed, accounting for 9.09% (*n* = 16) and 2.27% (*n* = 4), respectively. Rhinophyma was absent in most cases (84.7%, *n* = 149), while mild rhinophyma was observed in 11.9% (*n* = 21). Moderate and severe rhinophyma were rarely reported in 2.84% (*n* = 5) and 0.57% (*n* = 1), respectively. Regarding ocular involvement, most participants (72.2%, *n* = 127) reported no irritation, whereas mild symptoms were present in 21.0% (*n* = 37). Moderate and severe irritation were relatively uncommon, reported by 5.68% (*n* = 10) and 1.14% (*n* = 2) of patients, respectively (Figure 1).

**Table 2:** Self-assessment of rosacea severity.

Variables	*N* = 176
CAT score	5.00 [3.00;7.00]
Papules	
1	114 (64.8%)
2A	42 (23.9%)
3B	16 (9.09%)
4C	4 (2.27%)
Rhinophyma	
None	149 (84.7%)
Mild A	21 (11.9%)
Moderate B	5 (2.84%)
Severe C	1 (0.57%)
Ocular irritation	
None	127 (72.2%)
Mild A	37 (21.0%)
Moderate B	10 (5.68%)
Severe C	2 (1.14%)

Categorical variables were summarized using *n* (%), and continuous variables were summarized using median [IQR].

**Figure 2:**
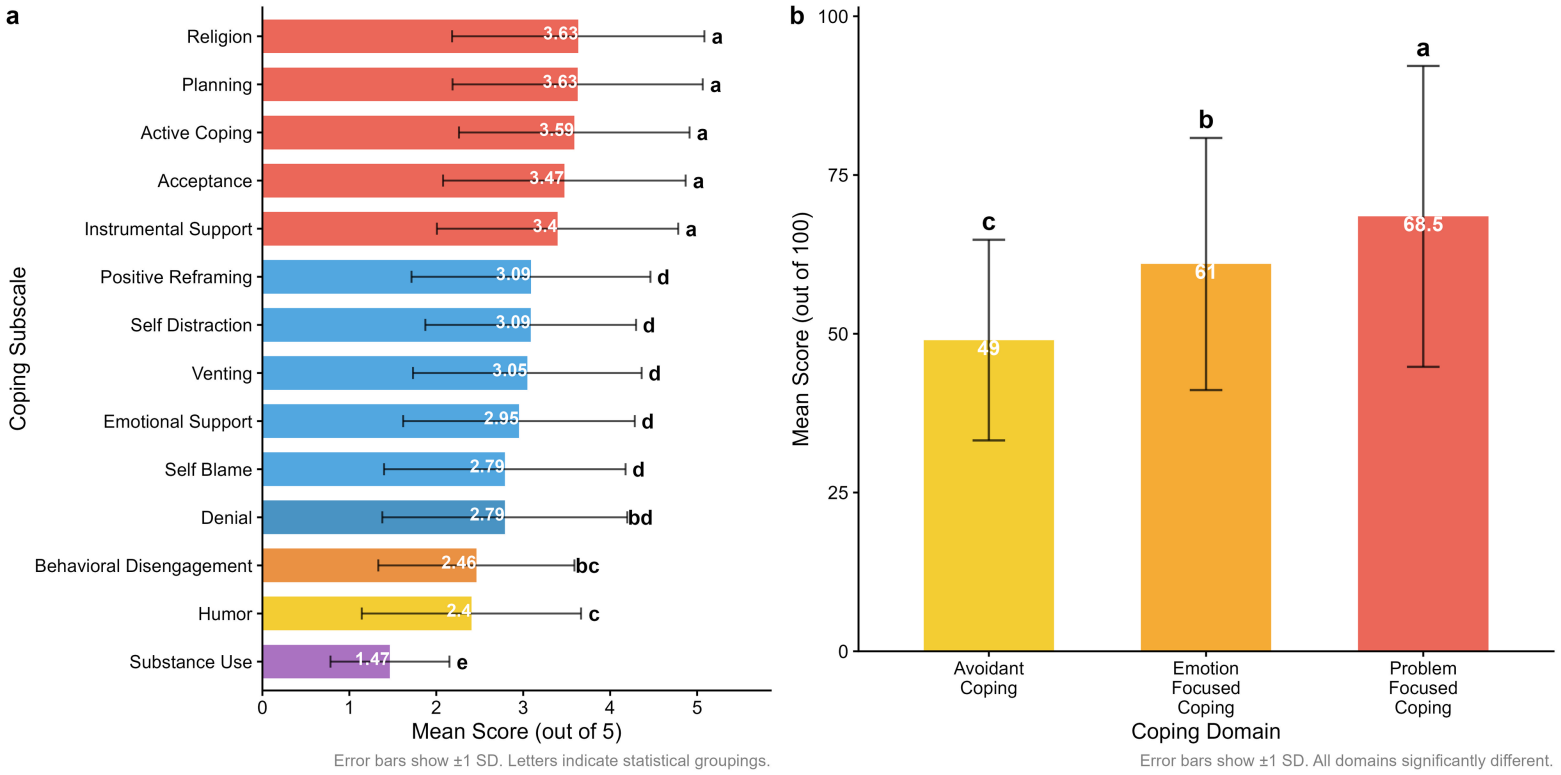
**(A)** Individual coping strategies usage patterns. **(B)** Overall coping domain effectiveness. **(A)** Individual coping strategy usage patterns among study participants were scored on a 0–5 scale. Error bars represent ±1 SD. Post-hoc pairwise comparisons were performed using paired t-tests with the Bonferroni correction. Letters indicate homogeneous subsets where strategies sharing the same letter are not significantly different (*p* > 0.05). **(B)** Comparison of mean scores across the three primary Brief-COPE coping domains. Error bars represent ±1 SD. Post-hoc analysis used paired *t*-tests with Bonferroni correction to identify significant differences between domains (*p* < 0.05). Letters indicate statistical groupings. The scales were standardized to a 0–100 scale for comparability across domains.

Table 3 presents the descriptive statistics for the included COPE-Brief subscales and domains. Among all Brief-COPE subscales, planning (mean = 3.63), religion (3.63), active coping (3.59), acceptance (3.47), and instrumental support (3.40) exhibited the highest mean scores and constituted a statistically homogeneous subset. While these subscales had significantly higher mean scores than the remaining subscales, no significant differences were found among them. A second subset included positive reframing (mean = 3.09), venting (3.05), self-distraction (3.09), emotional support (2.95), self-blame (2.79), and denial (2.79). The mean scores of these subscales did not differ significantly from each other but were significantly lower than those of the first group and significantly higher than the remaining subscales. Notably, denial statistically overlapped with behavioral disengagement (2.46). Humor (mean = 2.40) and behavioral disengagement (2.46) formed a distinct third group, with significantly lower scores than the previous two subsets. Substance use had the lowest mean score (1.47) and was substantially lower than all other subscales (Figure 2A).

**Table 3:** Descriptive statistics for the included COPE-Brief subscales and domains.

Variable	Mean	SD	Median	Q1	Q3
Subscale					
Active Coping Score	3.59	1.33	3.75	2.50	5.00
Instrumental Support Score	3.40	1.39	3.75	2.50	5.00
Positive Reframing Score	3.09	1.37	3.12	1.88	4.38
Planning Score	3.63	1.44	4.06	2.50	5.00
Emotional Support Score	2.95	1.33	3.12	1.88	3.75
Venting Score	3.05	1.31	3.12	1.88	4.38
Humor Score	2.40	1.26	1.88	1.25	3.12
Acceptance Score	3.47	1.40	3.75	2.50	5.00
Religion Score	3.63	1.45	3.75	2.50	5.00
Self-Blame Score	2.79	1.39	2.50	1.25	3.75
Self-Distraction Score	3.09	1.21	3.12	1.88	4.38
Denial Score	2.79	1.41	2.50	1.25	3.91
Substance Use Score	1.47	0.68	1.25	1.25	1.25
Behavioral Disengagement Score	2.46	1.13	2.50	1.25	3.12
Domain					
Problem-Focused Coping	68.48	23.69	75.00	56.25	87.50
Emotion Focused Coping	60.98	19.85	62.50	49.48	75.00
Avoidant Coping	49.01	15.80	50.00	37.50	59.38

Pairwise comparisons were conducted using the paired *t*-test post-hoc test. Homogeneous subsets were identified using compact letter displays. Q1: 25% percentile; Q3: 75% percentile.

A comparison of the three Brief-COPE domains revealed significant differences in patients’ average use of coping strategies. Problem-focused coping had the highest mean score (68.48), followed by emotion-focused coping (60.98); avoidant coping had the lowest mean score (49.01). These differences were statistically significant, indicating that participants utilized problem-focused strategies more frequently than emotion-focused or avoidant strategies. The internal consistency of the three domains, as measured by Cronbach’s alpha (α), was 0.91 for problem-focused coping, 0.88 for emotion-focused coping, and 0.75 for avoidant coping, demonstrating good reliability across the domains (Figure 2B).

Table 4 presents the correlation between Brief-COPE domain scores and the severity of rosacea. The results show that disease duration was positively correlated with all three coping strategies and rosacea severity. The strongest correlation was observed between disease duration and emotion-focused coping (r = 0.323, *p* < 0.001), followed by avoidant coping (r = 0.268, *p* < 0.001) and problem-focused coping (r = 0.257, *p* < 0.001). A positive correlation was also identified between disease duration and the CAT score (r = 0.317, *p* < 0.001). Conversely, age did not show a significant correlation with any of the three coping domains (r = 0.090–0.097, *p* > 0.05) or with the CAT score (r = 0.011, *p* > 0.05).

**Table 4:** Correlation analysis between Brief-COPE domain scores and rosacea severity.

Variables	Age	Duration of disease	Problem-focused coping	Emotion focused coping	Avoidant coping
Age					
Duration of disease	0.267^***^				
Problem-Focused Coping	0.090	0.257^***^			
Emotion Focused Coping	0.097	0.323^***^	0.839^***^		
Avoidant Coping	0.077	0.268^***^	0.645^***^	0.765^***^	
CAT score	0.011	0.317^***^	0.438^***^	0.474^***^	0.351^***^

Correlation Analysis was performed using Pearson’s method with listwise deletion. ***p < 0.001.

Table 5 shows the comparison of Brief-COPE domain scores across sociodemographic characteristics. The post-hoc analysis revealed significant differences in domain scores across educational and employment groups. For problem-focused coping (PFC), individuals with a high school education or less had significantly lower mean scores (55.4 ± 24.6) compared to those with a bachelor’s degree (71.5 ± 22.8) or postgraduate education (71.1 ± 21.6). However, no significant difference was observed between the bachelor’s and postgraduate education groups.

**Table 5:** Comparison of brief-COPE domain scores across sociodemographic characteristics.

Variable	Level	PFC	EFC	AC
Gender	Female	68.6 ± 24.2	61.3 ± 20.1	48.9 ± 16.1
	Male	67.9 ± 22	59.8 ± 19.2	49.5 ± 14.6
	*p*-value	0.865	0.684	0.841
Nationality	Non-Saudi	70.6 ± 23.7	62.5 ± 18.2a	51.5 ± 16.1
	Saudi	68.1 ± 23.7	60.7 ± 20.2a	48.5 ± 15.8
	*p*-value	0.600	0.661	0.373
Residence	Central region	66.7 ± 24.9	58.4 ± 20.8a	47.4 ± 16.3
	Eastern region	68.1 ± 23.1	59.4 ± 19a	49.7 ± 12.2
	Northern region	71.7 ± 23.4	61.7 ± 18.3a	42.7 ± 11.4
	Southern region	75.4 ± 22.3	69.7 ± 14.3a	54.2 ± 14.4
	Western region	68.4 ± 22.3	63.3 ± 20.3a	52.3 ± 17.5
	*p*-value	0.701	0.251	0.142
Marital status	Divorced	56.2 ± 44.2	38.5 ± 19.2	29.7 ± 6.6
	Married	67.5 ± 23.7	60.5 ± 20.9	48.5 ± 15.7
	Single	68.8 ± 23.6	61.5 ± 19.1	49.4 ± 15.7
	Widowed	87.5 ± 12.5	71.5 ± 16.8	62.5 ± 18.8
	*p*-value	0.459	0.322	0.150
Education	Bachelor’s degree	71.5 ± 22.8 b	62.8 ± 19.8	50.1 ± 15.4
	High school or less	55.4 ± 24.6 a	55.1 ± 21.1	43.6 ± 16.1
	Postgraduate	71.1 ± 21.6 b	59.8 ± 17.1	50.8 ± 16.4
	*p*-value	**0.002**	0.142	0.095
Employment	Employed	72.2 ± 19.7 b	64.6 ± 18.3 b	52.5 ± 15.7 b
	Housewife	65.9 ± 27.1 ab	59.8 ± 23.6 ab	48.2 ± 16.2 ab
	Student	58 ± 25.6 a	52.8 ± 19.6 a	43.3 ± 15.8 a
	Unemployed	78.6 ± 19.3 b	66.9 ± 16 b	51.1 ± 13.7 ab
	*p*-value	**<0.001**	**0.003**	**0.016**
Monthly income	<5,000	66.7 ± 23.5a	62.1 ± 19.2a	48.8 ± 14.1a
	>10,000	66.2 ± 24.4a	57.8 ± 20.6a	46.8 ± 16.4a
	5,000–10,000	73.5 ± 22.3a	65.3 ± 18.4a	52.7 ± 15.7a
	*p*-value	0.186	0.095	0.101

PFC = Problem-Focused Coping, EFC = Emotion-Focused Coping, AC = Avoidant Coping. Analysis was conducted using One-way ANOVA, followed by post-hoc comparisons using unpaired t-tests for all variables except gender, which was analyzed using an unpaired t-test. Post-hoc analysis was performed using Bonferroni adjustment. Homogeneous subsets were identified using compact letter displays, where means not sharing the same letter are significantly different (*p* < 0.05). Bold value means *p* < 0.05.

Regarding employment, students had significantly lower problem-focused coping (PFC) scores (58.0 ± 25.6) compared to employed individuals (72.2 ± 19.7) and unemployed individuals (78.6 ± 19.3). Housewives (65.9 ± 27.1) exhibited intermediate scores and did not differ significantly from the employed or unemployed groups. A similar pattern was observed for emotion-focused coping (EFC) and avoidant coping (AC), with students displaying the lowest mean scores (52.8 ± 19.6 for EFC and 43.3 ± 15.8 for AC), which were significantly lower than those of employed participants (64.6 ± 18.3 for EFC and 52.5 ± 15.7 for AC). Housewives showed intermediate values for both EFC and AC, with no significant differences between them and the other groups. No statistically significant differences were observed across gender, marital status, income, nationality, or region, as indicated by the absence of compact letter assignments for these categories.

## 4 Discussion

The current study found that adaptive and problem-focused coping strategies, particularly religion (M = 3.63), planning (M = 3.63), and active coping (M = 3.59), were the most frequently employed by patients with rosacea in Saudi Arabia. These three strategies formed a statistically homogeneous subset (*p* < 0.05). They significantly outperformed avoidant and emotion-focused coping methods, suggesting that rosacea patients in this context prefer constructive approaches to managing chronic stress. These results are consistent with broader research indicating that adaptive coping fosters better psychosocial outcomes in chronic dermatological conditions (4). Notably, religion’s prominence as a coping mechanism may reflect cultural norms in Saudi Arabia, where it is consistently the top strategy across various populations, including medical professionals, and during national stressors such as the COVID-19 pandemic (25–28). In other dermatological settings, such as psoriasis, patients’ greater reliance on adaptive strategies has been correlated with better illness acceptance and a willingness to seek care (29). These parallels highlight the cultural significance of religion and planning in Saudi Arabia, reinforcing the notion that effective coping strategies lead to enhanced psychosocial outcomes.

Rosacea’s chronic, visible nature imposes a considerable psychosocial burden, with patients frequently reporting embarrassment, reduced self-esteem, social withdrawal, and heightened anxiety (30, 31). These emotional challenges are compounded by stigmatization, which has been shown to exacerbate depression and lower quality of life across multiple domains, particularly social functioning (32, 33). Our finding that religion, planning, and active coping were the most frequently employed strategies among Saudi rosacea patients aligns with evidence that constructive coping approaches such as meaning-making through spirituality can buffer the adverse effects of visible skin conditions and foster resilience (30, 32). Conversely, maladaptive strategies, including avoidance and denial, have been linked to poorer psychosocial adjustment, higher distress, and reduced treatment satisfaction (33). Moreover, recent research suggests that illness perception plays a pivotal role in shaping quality of life outcomes in rosacea, with more severe disease correlating with greater psychosocial impairment (34). By reinforcing adaptive coping, particularly problem-focused and culturally congruent strategies, dermatological care may not only enhance emotional well-being but also improve long-term adherence and clinical outcomes in this patient group (31).

From a theoretical perspective, these coping patterns align with Lazarus and Folkman’s stress-coping model, which suggests that problem-focused coping is particularly effective when individuals perceive the stressor as modifiable. In the case of chronic rosacea, factors such as skincare routines, medication adherence, and trigger avoidance are controllable, prompting patients to adopt planning and active coping strategies. This finding mirrors other research on chronic diseases. For instance, research has found that problem-focused coping was most frequently used by patients with psoriasis, multiple sclerosis, and kidney dialysis or transplant recipients, while avoidant coping was the least common (35). Similarly, another study noted that chronic obstructive pulmonary disease (COPD) patients employing problem-focused strategies reported higher self-esteem and lower depression compared to those using avoidant methods (36). Furthermore, problem-focused coping was linked to better lung function in COPD (FVC%; r = 0.400, *p* = 0.035), highlighting a connection between psychological and physiological health (37).

Global literature consistently shows that emotion-focused and avoidant coping styles tend to worsen distress. These styles have been found to mediate the adverse effects of disease severity on perceived illness intrusiveness in cardiopulmonary patients (38). Likewise, wishful thinking, self-blaming, and avoidance have been identified as predictors of poor psychosocial adjustment and increased depression (39).

Beyond the general preference for problem-focused coping, our post-hoc analysis revealed two statistically distinct clusters of coping strategies among rosacea patients. The high-scoring, adaptive cluster included religion, planning, active coping, acceptance, and instrumental support, while the low-scoring, maladaptive cluster encompassed behavioral disengagement, substance use, and humor (*p* < 0.05). This apparent dichotomy between adaptive and nonadaptive coping has been well-documented in chronic illness research. For example, a study categorized fibromyalgia patients into resilient, adaptive, vulnerable, and maladaptive groups, with resilient patients *demonstrating* high levels of problem-focused and emotion-focused coping, along with low disability. In contrast, maladaptive subgroups exhibited greater disability and catastrophizing (40). In addition, “adaptive” and “nonadaptive” coping clusters have been identified among chronically ill individuals, with the latter group experiencing lower life satisfaction and heightened psychological distress (41). Parallel studies have extended these findings to caregivers and psychiatric populations; for instance, Sirois et al. found that self-compassion promoted positive reframing, acceptance, and reduced reliance on disengagement and self-blame, resulting in lower stress levels in patients with inflammatory bowel disease and arthritis (42).

These coping clusters represent distinct coping profiles and impact clinical and psychosocial outcomes. (43) found that avoidant coping in caregivers of children with chronic illnesses was associated with increased depression, while distraction strategies were linked to better emotional well-being. Similarly, (44) observed that physical activity as an adaptive strategy alleviated both mental and physical burdens among older adults with chronic pain, while resting and guarding negatively impacted quality of life (QoL). These findings emphasize the necessity for targeted interventions that strengthen adaptive coping and minimize maladaptive tendencies (42–46). Potential interventions to reinforce adaptive coping in rosacea patients include structured psychoeducational programs, culturally sensitive cognitive behavioral therapy (CBT), and guided support groups that address disease-specific emotional distress. Incorporating spiritual counseling and social support frameworks that align with Saudi Arabia’s cultural values may further enhance patient outcomes. Personalized coping assessments can also help tailor interventions to individual needs.

Furthermore, the current study found a positive correlation between disease duration and all coping domains, with emotion-focused coping showing the strongest correlation (r = 0.323, *p* < 0.001). Longer disease duration was also significantly correlated with higher rosacea severity (CAT score; r = 0.317, *p* < 0.001), suggesting that patients may expand their coping strategies as the disease progresses, possibly adopting more emotion-focused or avoidant methods over time. Education and employment status were identified as significant sociodemographic determinants of coping effectiveness. Participants with higher educational levels (bachelor’s or postgraduate degrees) scored 14.2% higher on problem-focused coping (PFC) compared to those with only secondary education (*p* < 0.05). Students consistently scored the lowest across all coping domains.

In line with Nurhayati et al., who found that higher education predicted greater use of adaptive coping (β = 0.97, *p* = 0.004) in breast cancer patients, thereby improving their quality of life (47). Similarly, Cheng et al. found that higher educational levels in Chinese patients with multiple chronic conditions were linked to better physical (β = 0.21, *p* < 0.01) and mental QoL (β = 0.19, *p* < 0.05), mediated by adaptive coping (48). In Pakistan, less-educated hepatitis C patients scored 33% higher in religious-spiritual and emotion-focused coping, while university-educated patients relied more on problem-focused coping and instrumental support (49). Similar patterns have also been observed in chronic pain populations (50).

Chronic illnesses can disrupt employment; a Dutch registry study (*N* = 667,002) found that chronic illness delayed job entry by up to 10.4 months, potentially affecting coping strategies (51). In a UK/US comparison, (52) found that employment rates among young adults with end-stage kidney disease were higher in the UK (60%) than in the US (41%), highlighting differences in systemic support. Students, in contrast, often lack financial stability and institutional support, leading to increased reliance on emotional or avoidant coping (53).

Our findings emphasize the dominance and effectiveness of problem-focused and adaptive coping strategies among Saudi rosacea patients, particularly religion, planning, and active coping. These strategies significantly outperformed avoidant and emotion-focused methods (*p* < 0.05), a pattern consistent with global literature on coping strategies in chronic disease (35, 54). Post-hoc clustering revealed a clear distinction between high-scoring adaptive strategies (e.g., acceptance, instrumental support) and low-scoring maladaptive strategies, such as behavioral disengagement and substance use, consistent with well-established classifications of “adaptive” versus “nonadaptive” (40, 41). Moreover, our data indicate that longer disease duration is associated with a broader range of coping behaviors, with education and employment *emerging as* key enablers of adaptive coping. In light of these findings, culturally tailored interventions must be developed that foster problem-focused coping, integrate social and spiritual support, and address sociodemographic factors, particularly among students and those with lower educational levels. These findings highlight the importance of integrating psychosocial considerations into dermatological care. Adaptive coping mechanisms such as active coping and planning can reduce psychological distress and improve patients’ quality of life. At the same time, the presence of maladaptive strategies such as denial or self-blame may hinder recovery, underscoring the need for timely psychological assessment and culturally tailored support services. By translating these insights into clinical practice, healthcare providers can design more effective, patient-centered support programs. Public health efforts should also aim to raise awareness about the emotional toll of skin conditions and empower patients with tools to manage both the physical and psychological burden of rosacea.

Several limitations should be considered when interpreting the findings of this study. First, its cross-sectional design limits the ability to infer causality between coping styles and long-term psychosocial consequences. Second, reliance on self-reported data introduces the potential for recall bias and social desirability bias, although validated and standardized instruments were employed to minimize these effects. Third, the sample exhibited a notable gender imbalance, with females comprising 79.5% of the participants. This overrepresentation may limit the generalizability of the findings to broader, more gender-balanced populations. Future research should include more diverse and representative samples to enhance external validity. Additionally, the scoring assessments (CEA and CAT) were adapted for self-assessment using visual guides. At the same time, this enabled broader participation, it may introduce variability in interpretation compared to clinician-administered scoring.

Moreover, this study did not assess the quality of life (QoL) of patients, which represents an important psychosocial outcome in chronic dermatological conditions. Future studies are recommended to incorporate validated QoL instruments better to capture the lived experience of individuals with rosacea.

## 5 Conclusion

In conclusion, patients with rosacea in Saudi Arabia predominantly engage in adaptive, problem-focused coping strategies, with religion, planning, and active coping emerging as the leading methods, which significantly outscore emotion-focused or avoidant methods. While these findings may reflect cultural and contextual influences, further cross-national research is needed to confirm this. Our results suggest that interventions supporting problem-focused approaches and acknowledging patients’ spiritual and social resources may be beneficial. By recognizing the role of coping style in both psychological and functional domains, healthcare providers can tailor their support to help rosacea patients attain better long-term outcomes.

## Data Availability

The original contributions presented in the study are included in the article/supplementary material, further inquiries can be directed to the corresponding author.
